# Before and During the First COVID-19 Surge: Work Conditions, Burnout, and Mental Health Among Resident Physicians in a Department of Psychiatry in the USA

**DOI:** 10.1007/s40596-023-01844-z

**Published:** 2023-08-27

**Authors:** Alpna Agrawal, Kazandra De La Torre, Conisha Cooper, Jeremy Flores, Karen Miotto, Kenneth Wells, Elizabeth Bromley, Elizabeth M. Yano, Jonathan Heldt, Enrico G. Castillo, Katrina DeBonis

**Affiliations:** 1 Geffen School of Medicine at University of California, Los Angeles Medical Center, Los Angeles, CA USA; 2 VA Greater Los Angeles Healthcare System, Los Angeles, CA USA; 3 UCLA Fielding School of Public Health, Los Angeles Medical Center, Los Angeles, CA USA

**Keywords:** COVID-19, Resident physicians, Occupational and mental health risks

## Abstract

**Objective:**

Resident physicians are critical frontline workers during pandemics, and little is known about their health. The study examined occupational and mental health risks among US psychiatry residents before and during the first COVID-19 surge.

**Methods:**

Longitudinal data were collected from a cohort of US psychiatry residents at one academic medical center in October 2019, before the pandemic, and April 2020 after the initiation of a state-level stay-at-home order. Primary outcome measures were psychological work empowerment, defined as one’s self-efficacy towards their work role, and occupational burnout. A secondary outcome was mental health. In May and June 2020, resident engagement sessions were conducted to disseminate study findings and consider their implications.

**Results:**

Fifty-seven out of 59 eligible residents participated in the study (97%). Half the study sample reported high burnout. From before to during the first COVID-19 surge, psychological work empowerment increased in the total sample (*p* = 0.03); and mental health worsened among junior residents (*p* = 0.004), not senior residents (*p* = 0.12). High emotional exhaustion and depersonalization were associated with worse mental health (*p* < 0.001). In engagement sessions, themes related to residents’ work conditions, COVID-19, and racism emerged as potential explanations for survey findings.

**Conclusions:**

The study is exploratory and novel. During early COVID, psychiatry residents’ well-being was impacted by occupational and societal factors. Postpandemic, there is a growing psychiatrist shortage and high demand for mental health services. The findings highlight the potential importance of physician wellness interventions focused on early career psychiatrists who were first responders during COVID.

Public health disasters impact the mental health of physicians working on the frontlines. Early COVID was challenging for resident physicians—a key constituency of the frontline workforce. Scant research is available on psychiatry trainees’ work conditions and health during COVID, a public health disaster that uniquely highlighted the mental health burden in the USA [1–3]. Psychiatry trainees played a critical role in health systems’ response to mental health service demands during COVID, but little is known about their work experiences and psychological health during this period. The current study assessed occupational and mental health risks before and during the first COVID-19 surge among psychiatry residents at one academic medical center in the USA. The study’s goal was to identify future areas of focus in physician wellness, particularly among early career physicians in the medical field of psychiatry where workplace shortages are projected [4].

## Methods

The current study was initiated pre-pandemic in collaboration with the adult psychiatry residency program director. The study purpose was to assess educational and training priorities of a psychiatry training program, such as residents’ work conditions, professional development, and well-being. Physician leaders who were content experts in physician wellness and community research were consulted during study development and implementation. Study participants were psychiatry residents enrolled in a training program at an academic institution and recruited by email via an anonymous survey link distributed through Qualtrics®. Participation was voluntary and anonymous. During recruitment, all residents were provided information on support services offered through the Department of Psychiatry and institution broadly.

Validated measures were used to examine resident physicians’ occupational and mental health outcomes. Demographic information such as race/ethnicity, age, and gender were not collected to ensure participant anonymity in a small, worksite, study sample [5]. Surveys were administered over a 3- to 4-week period before (i.e., October 2019, time 1) and following the first state-issued stay-at-home order due to rapidly rising COVID-19 cases (i.e., April 2020, time 2).

Psychological work empowerment was assessed by the Psychological Work Empowerment Scale, a 12-item Likert scale measure. Higher scores indicate higher psychological work empowerment [6]. Occupational burnout over the last 3 months was measured by the Maslach Burnout Inventory (MBI)-Human Services Survey (MBI-HSS), a 22-item scale with three subscales: emotional exhaustion (EE), depersonalization (DP), and personal accomplishment (PA). High burnout was defined as EE scores ≥ 27 and/or DP scores ≥ 13 [7]. Self-reported mental health over the last 4 weeks was assessed by the Mental Composite Scale (MCS) Short-Form 12, version 1. Higher scores indicate better mental health, and a score change ≥ 5 points is considered clinically significant [8].

Resident engagement sessions designed to disseminate survey findings in a context that allowed for an interactive, two-way dialog were conducted [9]. All residents were invited to participate in either or both sessions held in May and June 2020 following survey administration. Three residents volunteered to serve as facilitators. Each virtual 1-h session consisted of (1) a brief presentation of study findings; (2) small group discussion on findings; and (3) return to the larger group to review small group discussion points. In small groups, facilitators asked residents to comment on factors they believed contributed to study findings and potential next steps. Qualitative notes summarized discussion points and feedback from participants. University institutional review board approved the survey study with a waiver of signed consent and exemption was granted for the anonymous, qualitative data from resident engagement sessions.

In study analyses, mean scores and outcome proportions were computed. Mean scores for study measures at time 1, time 2, and change between time points were estimated using linear mixed models by restricted maximum likelihood estimation (REML) producing average marginal effect scores. Linear mixed modeling under REML was employed to estimate the association between MBI subscales and mental health adjusting for covariates. Stata SEv16.1 was used for all analyses, and type-I error was set at 0.05. Qualitative data from session notes were organized into themes and reviewed by participants and facilitators for accuracy.

## Results

The total study sample included 57/59 (97%) residents who participated at time 1 or 2. Survey response rates were 81% (48/59) at time 1 (over 3 weeks) and 86% (51/59) at time 2 (over 2 weeks). Distribution of respondents by junior (i.e., PGY-1, PGY-2) and senior (i.e., PGY-3, PGY-4) training levels was comparable at time 1 (42% and 58%, respectively) and time 2 (53% and 47%, respectively) (*p* > 0.05).

The prevalence of high burnout among resident physicians was 48% at time 1 and 51% at time 2 (Table 1). Residents’ overall mean MCS score (over time 1 and 2) was 41.0 (11.4), which is 9 points below the US population norm score of 50.

**Table 1:** Mean work empowerment, occupational burnout, and mental component score before and during the first COVID-19 surge among general psychiatry residents by training level, October 2019 and April 2020 (*n* = 48 at time 1; *n* = 51 at time 2)

	Time 1: Before first COVID-19 surge			Time 2: During first COVID-19 surge			
Measures	*N*	Mean/AME	SD	*N*	Mean/AME	SD	*p-*value
Work empowerment^1^							
Total sample	46	59.1	1.29	51	61.8	1.24	0.028
a. Junior residents (PGY1 and 2)	19	59.6	1.94	27	61.1	1.71	0.420
b. Senior residents (PGY3 and 4)	27	58.8	1.72	24	62.6	1.79	0.020
Training level × time							0.348
Maslach Burnout Inventory^2^							
Emotional exhaustion score							
Total sample	48	22.7	1.55	51	21.6	1.52	0.227
a. Junior residents (PGY1 and 2)	20	21.6	2.25	27	22.3	2.11	0.669
b. Senior residents (PGY3 and 4)	28	23.8	2.15	24	30.0	2.19	0.045
Training level × time							0.096
Depersonalization score							
Total sample	48	9.69	0.84	51	9.26	0.82	0.579
a. Junior residents (PGY1 and 2)	20	9.94	1.25	27	10.4	1.12	0.701
b. Senior residents (PGY3 and 4)	28	9.45	1.14	24	8.2	1.18	0.236
Training level × time							0.278
Personal accomplishment score							
Total sample	48	37.5	0.91	51	38.2	0.89	0.343
a. Junior residents (PGY1 and 2)	20	38.5	1.34	27	38.1	1.22	0.752
b. Senior residents (PGY3 and 4)	28	36.6	1.24	24	38.4	1.28	0.092
Training level × time							0.171
Mental composite summary score^3^							
Total sample	45	42.2	1.66	49	40.7	1.62	0.315
a. Junior residents (PGY1 and 2)	19	45.3	2.45	27	39.4	2.22	0.004
b. Senior residents (PGY3 and 4)	26	39.1	2.26	22	42.1	2.36	0.122
Training level × time							0.001

*Abbreviation: AME* average marginal effect, *SD* standard deviation

^1^Work empowerment refers to Psychological Empowerment at Work Scale score (range 12–84)

^2^Maslach Burnout Inventory consists of three subscales: emotional exhaustion (range 0–54), depersonalization (range 0–30), and personal accomplishment (range 0–48)

^3^Mental composite summary (MCS) score refers to MCS-12 score from SF-12v1 (range 0–100)

Psychological work empowerment among residents increased from before to during the first COVID-19 surge (time 1: 59.1 [1.29], time 2: 61.8 [1.24], *p* = 0.03). EE, DP, and PA scores did not change during this period (*p* > 0.05). No effects of training level on psychological work empowerment and burnout measures were observed by time (training level × time, *p* > 0.05) (Table 1).

No difference in mean MCS scores from before to during the first COVID-19 surge was observed (*p* > 0.05), but a training level × time interaction was observed (*p* = 0.001). Average marginal effects indicated MCS scores worsened among junior residents from 45.3 (2.5) to 39.4 (2.2) (*p* = 0.004) by clinically relevant levels based on the literature (> 9), while no change in senior residents’ MCS scores was observed (*p* > 0.05) (Table 1).

By burnout subscales, EE (*β* =  − 0.74, *p* < 0.001) and DP (*β* =  − 0.94, *p* < 0.001) were negatively associated with MCS scores adjusted for training level × time. PA was positively associated with MCS scores adjusted for training level × time (*β* = 0.73, *p* < 0.001).

Resident engagement sessions included participation from 22 resident physicians in May 2020 and 28 resident physicians in June 2020. Themes related to resident work conditions, COVID-19, and racism emerged during these discussions (Table 2). Participants’ reflections highlighted that junior residents experienced lower work autonomy, higher acuity caseload, and greater cohort fluctuations (e.g., residents leaving the program) than senior residents, potentially contributing to their worsening occupational health and mental health. Session participants shared that during the pandemic’s early stage junior residents also confronted greater uncertainty at work. Junior residents in these sessions reported moral distress when discharging and transferring patients due to their insurance status who were, in large part, racial/ethnic minorities as well. While residents indicated that such incidents of moral distress were not new, they expressed feeling heightened distress in the context of the pandemic, workplace stressors, and national events highlighting racism in the USA. Residents noted that after George Floyd’s death (May 25, 2020) and the deaths of others in protests, residents encountered silence or insensitivity from attendings regarding these events. Participants provided examples suggesting junior residents were more likely to have these experiences since they worked in multidisciplinary teams where such experiences were more pronounced, while senior residents primarily work in outpatient settings independently, not in a team setting.

**Table 2:** Themes and future directions that emerged during engagement sessions with general psychiatry residents on survey findings during first COVID surge, May 2020 and June 2020

A. Discussion points related to study findings on worsening mental health among junior residents, first and second year trainees, during first COVID surge	
1. General work conditions	Examples
• Lack of work autonomy	Limited flexibility in schedule, long work hours
• Changes in cohort	Members of class leaving training program
• High service burden	Reduced team size resulting in higher caseload
2. COVID-19 pandemic	Examples
• Lack of control and increased uncertainty	Shifting guidelines on appropriate PPE in inpatient setting
• Lack of safety at work due to exposure	Patients admitted to inpatient service without COVID testing
• Moral distress related to patient care	Unable to admit patients and discharging patients sooner than indicated
3. Racism	Examples
• Stress related to national events highlighting structural racism in the USA	Insensitivity and/or lack acknowledgement at the time of George Floyd’s murder and other incidents of police brutality from attendings on service
• Moral distress related to patient care highlighting structural racism in medicine	Unable to admit patients of color based on insurance status and transferring patients to hospitals with potentially insufficient mental health services and COVID safety precautions
B. Future directions	Examples
1. Increased communication with faculty	Regular communication and check-ins between faculty and residents promoting dialog
2. Increased inter-class dialog	Opportunities to discuss issues between classes to enhance collegiality and mitigate transitions between training years
3. Protection of class morale	Class-specific process groups during protected time such as resident didactics
4. Increased exploration and address of discrimination experienced and witnessed by trainees	Collecting information on demographics and experiences related to discrimination in future surveys among residents

In discussions on future efforts to improve residents’ work and health, participants emphasized the importance of communication throughout the training program to help process difficult work situations. Following the engagement sessions, one resident shared they were “struck by how little formal inter-class discussion there is about emotional experiences, professionalization, individual development” and “how faculty experiences on rotations have generally only been on a technical feedback level.” To address experiences related to racism in medicine, residents advised that future surveys collect information on demographics, discrimination, and COVID-related stress. One resident communicated that by not having demographic information, “we don’t necessarily see how... changes [due to] COVID might be impacting residents of particular backgrounds.”

## Discussion

Study findings show that from before COVID-19 to during the first COVID-19 surge in one academic setting, mental health worsened among junior resident physicians, not senior residents. Resident engagement sessions suggested residents were impacted by work conditions, COVID-19, and national events highlighting racism in the USA. The study expands the field’s understanding of early career physicians, specifically resident physicians, during public health disasters. This is the first study to assess US psychiatry residents’ occupational and mental health risks before and during the first COVID-19 surge when mental health service demand increased and resident physicians in psychiatry were frontline responders in the inpatient and outpatient settings [10].

Junior residents’ worsened mental health from before to during the first COVID-19 surge potentially reflects the high demand/low control nature of their work, in general, that was exacerbated by the pandemic and significant national events [11]. During the study period, junior residents were confronted with exposure risk to COVID pre-vaccine and subject to sudden changes in their workflow as guidelines shifted daily in the inpatient setting. Junior residents, who are responsible for evaluating patients in the emergency department, provided care to patients with unknown COVID statuses and admitted patients prior to the development of safety policies such as universal patient COVID testing. In addition, unclear protocols regarding resident quarantine following exposure resulted in tension among junior resident teams. In comparison, senior residents in psychiatry transitioned to telemedicine early on in the pandemic and did not face the same workplace exposure risks and stressors as junior residents. While our findings were observed during the COVID-19 pandemic, it is possible these results are secondary to general resident work conditions and differences by training level. In psychiatry, junior residents primarily work on inpatient units, have substantial overnight, in-house call, rotate on services not in their specialty, and treat sicker patients; senior residents, on the other hand, are outpatient, work alone, and have fewer on-call responsibilities.

Study findings illustrate psychological work empowerment improved among psychiatry residents overall. This may be indicative of increased work competence resident physicians typically gain in the latter half of the academic year. These findings may also be related to increased work responsibilities resident physicians took on during COVID-19 resulting in increased job satisfaction and sense of purpose at work. For example, during the pandemic’s early phase, residents in the program, particularly senior residents, volunteered to collaborate with leadership to develop COVID-19 patient care guidelines and an emergency response plan. In addition, leadership engaged in proactive communication and collaboration with residents from the outset of the pandemic potentially contributing to residents’ enhanced sense of control and empowerment at work. These study findings highlight the utility of identifying ways to improve work empowerment during and outside of public health disasters. While burnout is a common target of interventions in physician wellness, psychological work empowerment is associated with, and can impact, important occupational health outcomes such as job strain, organizational commitment, and job performance [12].

In the current study, burnout prevalence during the first COVID-19 surge was greater among psychiatry residents in this study (38%) compared to US otolaryngology residents (30%) [13]. In addition, study findings that burnout was stable from before to during COVID-19 support previous research illustrating burnout was steady over a given year and multiple years of resident training [14]. This study is the first to examine occupational burnout among US residents before and during a pandemic, uniquely capturing the early, acute effects of a public health disaster. The association observed between the burnout subscales and mental health reinforces the importance of continuing to identify and intervene on drivers of burnout in physician wellness interventions post-pandemic.

In resident engagement sessions, themes related to residents’ work conditions, COVID, and racism that emerged are consistent with other research describing the dual impact of two pandemics, racism and COVID, on healthcare workers [15, 16]. The sessions highlighted the importance of fostering meaningful dialog across residency classes and with departmental leadership, assessing moral distress and discrimination in future research, and anti-racism education for faculty and trainees. They also informed steps residency leadership took to address the issues raised in the engagement sessions. Residents received protected time for therapeutic discussions on processing events related to the pandemic, and program leaders addressed moral distress and work conditions during resident evaluations.

The study has several strengths and limitations. A study strength is its design which enabled examination of validated measures prior to and during the pandemic while accounting for correlated observations between subjects. Other study strengths were the high response rate and application of validated measures not previously examined among physicians during public health crises. Study limitations are that the findings are not generalizable since they are based on a single specialty, at a single institution, and in a single country. Specific measures were not collected to reduce survey burden and maintain resident anonymity [5]. Survey measures administered at time 2 possibly underestimated associations observed since they were not specific to the COVID-19 period alone. Finally, the study lacked a comparison group, not uncommon in disaster medicine studies.

Overall, the study findings on occupational and mental health risks among early career physicians before and during COVID-19’s first acceleration phase are exploratory and novel. The study supports an emerging theme in physician wellness research that there is an urgent need for initiatives to address early career physicians’ work and health following public health disasters while strengthening physician health at the community, institutional, and culture levels [17]. Physician wellness programs focused on early career physicians who were first responders as trainees during COVID-19 may be critical to retaining this cohort of physicians and mitigate projected shortages [1, 18]. Furthermore, interventions tailored by medical specialty and training level may be more effective such as initiatives targeting early career physicians in psychiatry given the field’s current landscape in the post-pandemic era with the rise in suicides nationally, high demand for mental health services, and growing shortage of psychiatrists [19, 20].
